# Cartilage repair: A review of Stanmore experience in the treatment of osteochondral defects in the knee with various surgical techniques

**DOI:** 10.4103/0019-5413.65136

**Published:** 2010

**Authors:** S Vijayan, G Bentley, TWR Briggs, JA Skinner, RWJ Carrington, R Pollock, AM Flanagan

**Affiliations:** Joint Reconstruction and Cartilage Transplantation Unit, Royal National Orthopaedic Hospital, Brockley Hill, Stanmore, Middlesex, HA7 4LP, United Kingdom

**Keywords:** Cartilage injuries, cartilage repair, cartilage regeneration, autologous chondrocyte implantation

## Abstract

Articular cartilage damage in the young adult knee, if left untreated, it may proceed to degenerative osteoarthritis and is a serious cause of disability and loss of function. Surgical cartilage repair of an osteochondral defect can give the patient significant relief from symptoms and preserve the functional life of the joint. Several techniques including bone marrow stimulation, cartilage tissue based therapy, cartilage cell seeded therapies and osteotomies have been described in the literature with varying results. Established techniques rely mainly on the formation of fibro-cartilage, which has been shown to degenerate over time due to shear forces. The implantation of autologous cultured chondrocytes into an osteochondral defect, may replace damaged cartilage with hyaline or hyaline-like cartilage. This clinical review assesses current surgical techniques and makes recommendations on the most appropriate method of cartilage repair when managing symptomatic osteochondral defects of the knee. We also discuss the experience with the technique of autologous chondrocyte implantation at our institution over the past 11 years.

## INTRODUCTION

Primary Osteoarthritis (OA) of the knee increases with age throughout the human race and is commonly encountered by many clinicians in patients over 50 years of age. Presenting symptoms include severe pain, swelling and clicking of joints and many of these patients become candidates for total joint replacement. However, observations made in our unit have shown the appearance of a large cohort of young patients being referred with secondary and early onset primary OA, as a result of articular cartilage injury. Often these cases are due to misdiagnosis and poor management of predisposing conditions (mainly trauma). The debate regarding management of this group of patients persists. Established surgical methods of management have shown chiefly the formation of fibro-cartilage, which has poor resistance to shear forces. However, the development of autologous chondrocyte implantation (ACI) and its variants has shown the production of hyaline or hyaline-like cartilage in the treatment of symptomatic articular cartilage injuries, leading to improved function in the long-term. Hence we question the continued use of previous surgical repair methods. Based on current literature, this review will briefly discuss the pathology of such injuries in young patients and the various surgical methods currently available for the treatment of articular cartilage injuries. We also describe the experience with the technique of ACI and its variants over the past 11 years at our institution.

## METHODS

This review is based on our 11-year experience in treating patients with symptomatic osteochondral defects, with the various surgical methods of articular cartilage repair in a centre of excellence (Joint Reconstruction and Cartilage Transplantation Unit, Royal National Orthopaedic Hospital, Stanmore, United Kingdom). During this time, we have conducted several studies and will present our data. We have reviewed the current literature and included studies that have assessed such methods critically, including the clinical outcomes of randomized controlled trials, which have been used in the management of articular cartilage injury.

### Cartilage injury

**Figure 1:**
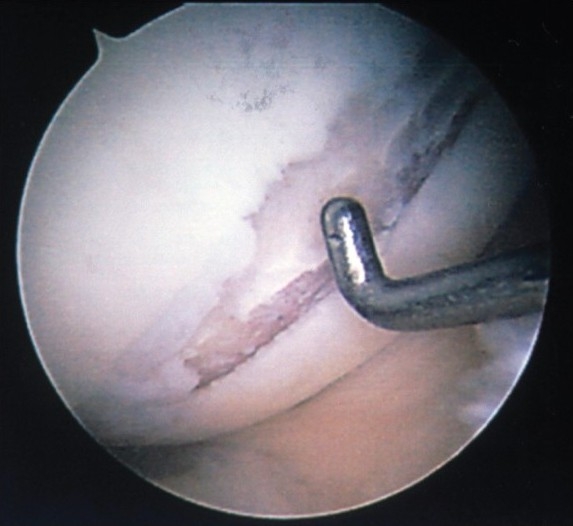
Arthroscopic photograph of an osteochondral defect of the medial femoral condyle showing exposure of subchondral bone

Joints are lined with smooth articular cartilage essential for low friction movement and shock absorption. Breakdown of this cartilage from trauma or disease is referred to as a chondral or if the underlying bone is involved, an osteochondral defect (OCD) [Figure 1]. In the knee, such defects can become symptomatic, resulting in severe pain and swelling of the joint,^1^ ultimately leading to the exposure of the underlying bone, causing pain, disability and eventually early onset OA. The natural intrinsic repair of articular cartilage lesions is very limited and highly dependent on age, depth, size, location and the nature of the injury.^2^ Consequently, cartilaginous injuries normally fail to heal spontaneously and larger lesions can result in a symptomatic degeneration of the joint leading to OA.^3^ Ageing also results in the reduced ability of chondrocytes to secrete matrix proteoglycans and collagen, which contributes to the degeneration of articular cartilage.^4^ Cartilage repair treatments are now based on trying to re-create and replace the normal hyaline articular cartilage.

### Incidence of localized cartilage lesions and OA

In the United Kingdom general practice, 1% of people aged over 45 years of age have a current clinical diagnosis of knee OA and 5% have a previously-made diagnosis of OA in another joint.^5^ Community-based studies have shown radiographic OA of the knee to be common. Recent studies show that 6% of adults aged 30 or more have frequent knee pain and radiographic OA.^6^

Twenty five per cent of all severe ligament and capsular knee injuries in young patients (aged 15 to 55 years), resulting in swelling of the knee, are associated with articular cartilage damage.^7^,^8^ One study has shown that of 31,516 knee arthroscopic procedures, in patients with a mean age of 43 years (range 1 year to 92 years), 63% were shown to have damage of the cartilage lining the knee when assessed by the International Cartilage Repair Society (ICRS) knee evaluation criteria.^7^ A more recent review of 993 consecutive arthroscopies in patients with a mean age of 35 years (range 10 year to 86 years), showed 655 (66%) having damage to the cartilage lining their knee.^9^ One long-term follow-up study reported on the natural history of minimally-symptomatic articular cartilage lesions in a group of 28 young athletes.^3^ At 14-year follow-up, 57% showed radiological evidence of reduction in joint space in the affected compartment,^3^ suggesting that cartilaginous lesions left untreated, may cause later symptoms and lead to early onset OA of the joint over time.

Given this information, it has become well-established that the number of cartilage repair procedures being performed each year is increasing,^10^ hence OCDs in younger patients should not be ignored and treatment should be more active. At present, there is no conclusive evidence in the literature to demonstrate that any cartilage repair procedure delays the onset of OA but symptomatic relief of symptoms occurs in 70 to 80%. However, there is still much debate as to what is the most successful surgical intervention when managing OCDs of the knee.

### Bone marrow stimulation techniques

Abrasion arthroplasty, drilling and micro-fracture are all forms of reparative “marrow-stimulating techniques.” Such methods aim to pierce the underlying subchondral bone of the defect, thereby inducing bleeding at the defect site and allowing for the formation of a blood clot. The clot contains pluripotent mesenchymal stem cells from the bone marrow, which have the potential to differentiate into fibrocytes. However, the resultant cartilage formed is fibro-cartilaginous with varied amounts of collagen Types I, II and III as opposed to the original hyaline articular cartilage.^11^ Fibro-cartilage cannot mimic the unique biochemical properties of hyaline articular cartilage, thereby failing to prevent further degeneration, leading to eventual breakdown of the repair tissue and the return of pain.^12^

### Abrasion arthroplasty (Debridement)

Cartilage surrounding symptomatic lesions is usually fibrillated and non-functional; hence surgical debridement involves the local excision of this entire area of unstable cartilage in the hope of new tissue formation from the bony base of the debrided lesion. Abrasion arthroplasty was described in the treatment of osteoarthritic knees prior to the development of total knee replacement^13^ but recent studies have shown conflicting conclusions,^14^,^15^ and more recently a Cochrane review has confirmed that it is not effective in the treatment of OA.^16^ Symptomatic and functional outcome improvement has been shown for five years post-treatment of medial femoral condylar articular cartilage defects, with significant decline afterwards.^17^ Hence, clinical practice involves the use of joint debridement combined with reparative techniques including micro-fracture to try to improve the surface tissue.^18^

### Drilling

Pridie et al. (1959) described the technique of subchondral drilling through exposed eburnated bone to stimulate cartilage repair in osteoarthritic knees.^19^ An animal study in 1976 showed that drilling OCDs in adult rabbit knees resulted in the formation of cartilage repair tissue, but the tissue deteriorated after 12 months.^20^ Drilling is known to cause thermal necrosis of the subchondral bone, as well as resulting in an uneven repair surface^11^ and for these reasons is not a favoured method of treatment.

### Micro-Fracture

Micro-fracture, a modification of drilling, is a single stage arthroscopic procedure developed by Steadman *et al*.^21^ in the 1980s. It involves the debridement of damaged articular cartilage down to the underlying subchondral bone-plate whilst preserving a stable perpendicular edge of healthy cartilage. Then, multiple holes are made in the bone in the base of the defect with a sharp awl with slight damage of the underlying bone plate. The defect is filled with a blood clot, allowing for repair by cells from pluripotential bone marrow cells.^22^ The environment created allows for the formation of new fibro-cartilaginous repair tissue.^22^ Despite micro-fracture not reproducing hyaline articular cartilage, fibro-cartilage repair has been shown to provide some symptomatic relief.^23^ However, the debate as to how effective and long-lasting micro-fracture is in patients with OCDs remains.

Several clinical studies have shown improvement in knee function in 70-90% of patients in the first year post treatment.^21^,^23^,^24^–^27^ Hunziker *et al*. suggested that micro-fracture shows clinical improvement for up to five years with rapid decline thereafter.^28^ More recently, Steadman *et al*. suggested that 80% of patients who underwent micro-fracture rated themselves as having clinical improvement seven years post-operatively and he found it most beneficial in patients under age 35 years.^26^ He further advocated its use in the treatment of symptomatic OCD in professional sportsman and from a total of 25 athletes, 76% returned to full sporting activity by the next season. However, this fell significantly to 36%, at a mean follow-up of 4.5 years, 29 supporting the results of Hunziker *et al*.^28^ This was further supported by Gobbi^24^ and Mithoefer *et al*.^30^ who reported significant decline in activity levels in high-level athletes 1-2 years after micro-fracture.

### Autologous Matrix-induced chondrogenesis (AMIC)

Steinwachs *et al*.(2008)^31^ recently reported the technique of AMIC. AMIC involves the joint use of the Chondro-Gide (Geistlich Biomaterials, Wolhausen, Germany) Type I / III collagen membrane as a scaffold over a defect treated by micro-fracture.^31^ Short-term results are encouraging, however, long term follow-up data is needed to substantiate preliminary findings.^32^

### Cartilage tissue-based therapy

#### Mosaicplasty (osteochondral autograft transplantation)

**Figure 2:**
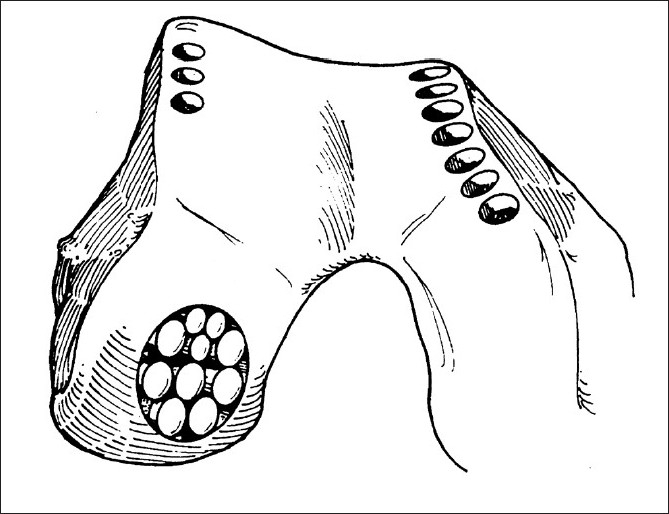
Line diagram illustrating the technique of Mosaicplasty (Reprinted with permission of the British Medical Journal)

Mosaicplasty was first reported in 1993 by Matsusue *et al*.^33^ Performed via an open operation, various sized cylindrical osteochondral plugs (usually mean 4.5 cm^2^) are taken from the periphery of both femoral condyles. These plugs are then placed into a pre-prepared OCD in a mosaic fashion [Figure 2]. Hangody *et al*. published a preliminary report on the technique in 1997^34^ and further follow-up studies on the same series.^35^,^36^ In their study, a total of 791 patients were treated and on average 86% of patients reported good/excellent results.^35^ However, this level of success has not been repeated and Bentley *et al*. showed rapid deterioration in all but the very small (0.5 cm^2^) defects in the short-term.^37^

Established limitations of the technique include technical difficulty,^38^ donor site morbidity,^34^ limited amount of donor tissue available, poor lateral tissue integration with native tissue, chondrocyte death from osteochondral plug impaction and the great difficulty in trying to recreate the smooth articular surface of the knee joint when fitting the plugs into the OCD. At best, mosaicplasty has been shown to produce islands of mature functioning hyaline articular cartilage surrounded by Fibro-cartilage filling the margins around the plugs, 35 which deteriorates over time leaving an intrinsic weakness in the repair system.^37^ A few good results have been reported,^39^,^40^ but mosaicplasty is less commonly used in current cartilage repair practice and is indicated only for very small lesions (< 1cm^2^).

### Cartilage cell-seeded therapies

#### Autologous chondrocyte implantation

Early experimental studies on animals by Bentley *et al*. first demonstrated the culture, storage and successful transplantation of articular and epiphysial chondrocytes into joint surfaces.^41^,^42^

**Figure 3:**
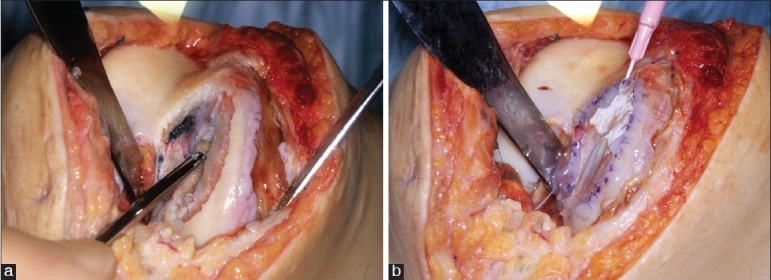
Operative photograph at the second stage of ACI (a) showing a large osteochondral defect in the medial femoral condyle. (b) showing the Chondro-Gide membrane sutured on the defect (Ch) and the injection of the cultured chondrocytes behind it

Autologous chondrocyte implantation (ACI), also referred to as autologous chondrocyte transplantation (ACT), was pioneered for clinical practice by Brittberg *et al*. (1994)^43^ and has given new hope in the treatment of symptomatic OCDs in young patients. It is a two-stage procedure in which a portion of a patient’s cartilage is harvested from a non weight-bearing portion of the knee arthroscopically. These cells are cultured and multiplied over a period of four to five weeks, before being implanted back into the OCD in the knee under a patch via an open operation.^43^ The first generation of ACI involved cells being injected beneath a periosteal patch (ACI-P) taken from either the tibia or femur,^43^ with second generation ACI using a collagen type I/III patch (ACI-C). An example of this collagen patch is Chondro-Gide (Geistlich Biomaterials, Wolhausen, Germany) [Figure 3]. Post-operatively, patients undergo an intensive eight-week physiotherapy rehabilitation program with a view to returning to full day-to-day activities within the first year, and are advised not to engage in high impact sport until a minimum of one year post-treatment. This technique proved to be the first application and coupling of bio-medically engineered cells in clinical Orthopedic Surgery. The results of ACI by Brittberg *et al*. at a mean follow-up of 39 months, in 23 patients, gave 70% good / excellent clinical outcome.^43^ Krishnan *et al*. reported from Stanmore that the ideal candidate for ACI was young (age 15 to 50 years), with a low body mass index, moderate pre-operative knee function, with symptoms lasting less than two years and having had fewer than two previous procedures on the affected knee.^44^

Our series also showed 88% of patients with ACI had good or excellent results at a mean follow-up of 1.7 years.^37^ At 11-year follow-up, a larger series of 51 patients showed consistently durable results, highlighting the greatly beneficial value of ACI in the treatment of OCD.^45^ Jones *et al*. also showed that at a 16-year follow-up, more than 80% of patients showed improved knee function with minimal complications.^46^ However, joint pathology such as axial mal-alignment, meniscal damage, ligamentous instability and patellar mal-tracking should be assessed prior to ACI in a staged or combined technique, thereby preventing an increased stress load being placed on the ACI graft site post-implantation.

The main drawback of ACI is the open surgical procedure (arthrotomy) needed to re-implant the cells in Stage 2 of the procedure, leading to scarring, muscle wasting and risk of reduced mobility. Our own experience and current practice favours the use of the Chondro-Gide membrane since it has a lower incidence of graft hypertrophy when compared with periosteum.^37^

### Matrix-assisted chondrocyte implantation

Matrix-assisted chondrocyte implantation (MACI) (Genzyme, Oxford, United Kingdom) is a later surgical technique of cartilage repair and is a third generation variant of conventional ACI. Instead of injecting cultured chondrocytes underneath a periosteal or collagen Type I / III cover, the cells are pre-loaded onto a commercially-produced porcine collagen patch. At the second stage of the operation, the patch is manually cut to cover the dimensions of the cartilage defect and held in place with tissue glue and, where necessary, sutures. At present there is limited data on the mid-to long-term follow-up success of such a technique. Behrens *et al*. published the clinical results of 25 patients two years after MACI, which showed functional knee scores improving post-operatively when compared to pre-operative scores.^47^ This is contrary to studies from our unit (Bartlett *et al*.^48^ ) and Manfredini^49^ who both reported no significant differences at two years in clinical outcome and knee assessment scores when comparing ACI to MACI in the treatment of symptomatic OCD of the knee. A recent study from our unit showed no clinical difference in results at two to five years with possibly better histological appearances after ACI.^50^ It is clear that longer-term comparative studies are needed to determine the therapeutic value of MACI when compared with other cartilage repair techniques, despite it being technically less demanding than conventional ACI.^48^

### Osteotomies

Osteotomies are advocated in patients presenting with early unicompartmental OA. Tibial osteotomy is the technique correcting alignment of the knee either by the removal of a segment of bone or by dividing the bone and inserting a wedge of bone graft or bone substitute. It is advocated in early unicompartmental OA and in the correction of mal-alignment of long bones with uneven weight-bearing especially at the knee. Several papers have been published on the beneficial use of osteotomies in combination with cartilage repair techniques.^51^,^52^ The most commonly used method is a high tibial osteotomy (resection of a wedge of the upper segment of the tibia) on the longer side or inserting of a wedge of bone (or substitute) on the shorter side. This “off-loads” the affected compartment and when used in conjunction with cartilage repair techniques techniques, has been shown to has been shown to help protect the graft site, thereby prolonging the longevity of the in-growing cartilage repair tissue. Currently no evidence exists that OCDs can heal spontaneously with osteotomy alone, therefore in younger patients, cartilage repair combined with osteotomy can provide the optimum environment needed to produce cartilage healing in the treatment of OCDs with femoro-tibial malalignment.

### Randomized comparative trials of articular cartilage repair techniques

Several randomized clinical trails have tried to provide an answer but, unfortunately, have shown conflicting results.

### ACI vs. mosaicplasty

One prospective study by Horas *et al*. comparing the two-year outcomes of 40 patients randomized to either moasicplasty or ACI for articular cartilage lesions of the femoral condyle found no significant difference in either method, with both providing symptomatic relief for patients.^39^ Bentley *et al* reported, a total of 100 patients, with a mean age of 31.3 years (range 16-49), an average defect size of 4.66cm^2^and a long history of a symptomatic OCD or chondral defect of the knee suitable for cartilage repair, which were randomized to either ACI or mosaicplasty.^37^ Of the total, 42 patients were randomized to mosaicplasty compared with 58 to ACI. Mean follow-up at 19 months (range 12-26) showed that 69% of the mosaicplasty patients had good/excellent results compared with 88% of the ACI group by assessment with the Modified Cincinatti^53^ and Stanmore scores.^54^ One-year check arthroscopy showed that histological results were much inferior with mosaicplasty patients; 34% demonstrated excellent or good repair compared with 82% of those randomized to ACI.^37^ We are in the process of following up these patients over an average 10-year period and preliminary results have shown a significant deterioration in those patients randomized to mosaicplasty and excellent to good long-term outcome of those randomized to ACI. Hence, we question the use of mosaicplasty in the treatment of OCDs of the knee in the short- and long-term.

### ACI vs. micro-fracture

A trial conducted in Norway between ACI (40 patients) and micro-fracture (40 patients) for symptomatic lesions of varied size (2-10cm^2^ ) of the femoral condyles reported similar outcomes in both groups after five years.^55^ However, the incidence of failure in the ACI group was less in patients whose histology was superior at a one-year biopsy.^55^

A Cochrane review published in 2006 by Wasiak and Villaneuva *et al*.^56^ reviewed several randomized trials including those of Bentley *et al*.^37^ Horas *et al*.^39^ and Knutsen *et al*.^24^ It came to the conclusion that at the present time there is no significant evidence that shows ACI to be better than any other method of cartilage repair. They concluded that larger numbers of randomized controlled trials, with longer periods of clinical follow-up is needed to resolve the current debate as to what is the most effective method of cartilage repair of OCD of the knee.

### MACI vs. micro-fracture

Basad *et al*. recently reported on the two-year clinical outcome of a group of patients involved in a randomized trail comparing MACI and micro-fracture.^57^ A total of 60 patients were involved, of which 40 received MACI and 20 micro-fracture for symptomatic, post-traumatic, single, isolated chondral defects of varied size (4-10cm^2^). He reported that MACI was far superior to micro-fracture in the treatment of articular defects of the knee over two years. He considered that MACI was superior to earlier cartilage cell-seeded therapies, as it was surgically less-demanding and invasive and therefore resulted in a reduced operative time.^57^

### Characterized chondrocyte implantation vs. Micro-fracture

Recently, different methods of cartilage repair have been developed. Characterized chondrocyte implantation (CCI) was reported by Saris *et al*. and involves a cell-surface marker profile allowing for the prediction of the likelihood to form hyaline-like cartilage ,*in vivo*.^58^ Saris *et al*. compared this technique of cell expansion and implantation with micro-fracture, finding superior histological results with similar clinical outcome scores at 3 years of CCI.^58^ Longer-term follow-up is needed to assess its true potential. This method has not been compared with standard ACI.

### Cohort studies

Several cohort, non-randomized studies have suggested that ACI^59^ or MACI^60^ give 80% of excellent or good results clinically for up to 9-10 years. However, these studies were not controlled, and whilst useful are not conclusive despite their results.

## DISCUSSION

The best method of treating OCDs of the knee by cartilage cell repair is a topic of much debate. Treatment of such defects depends mainly on the size and location of the defect as well as the patients’ age. Many clinicians have come to the conclusion that marrow-stimulation techniques such as micro-fracture and debridement should be reserved for smaller and well-contained lesions (< 1cm diameter). They suggest that micro-fracture can be undertaken as a first-line procedure in patients with OCD as it can be performed at the time of initial diagnostic arthroscopic assessment and does not compromise future cartilage repair surgery that may be needed. However, most studies have demonstrated that micro-fracture does not offer a long-term solution to OCDs of the knee,^24^,^28^,^29^,^30^ with the fibro-cartilaginous repair tissue generated deteriorating within two to five years. This is in contrast to larger lesions (> 1cm diameter), which are best treated with ACI and MACI, the technique of choice at our institute. Moreover, a recent study of 321 patients by Minas *et al*. confirmed that patients undergoing ACI secondary to previously-failed micro-fracture were three times more likely to fail with possible subchondral bone damage, than those patients treated with primary ACI.^61^ This implies that micro-fracture can have harmful effects on the subchondral bone-hypertrophy and cyst formation which will prejudice any later attempt at repair.

Our own experience has shown that OCDs greater than 1cm^2^, and those of the patella treated with mosaicplasty, fail in the short-term.^37^ However, re-alignment osteotomies combined with modern day cell-seeded therapies including ACI and MACI, may prove extremely beneficial in the management of uni-compartmental chondral lesions, therefore allowing for better long-term results in cartilage regeneration and preventing the early oset of OA and the need for total joint replacement in younger patients with its known higher complication rate.^62^

## THE FUTURE

At present we consider that the future of cartilage repair and regeneration lies in the realms of chondrocyte transplantation, in which methods such as ACI in the long-term have been shown to produce symptomatic pain relief, improved function and clinical outcome in patients over an 11-year period.^45^ Our own research has shown that the results of ACI are far superior in younger patients, with relatively good pre-operative knee function, who have had fewer than two previous procedures for the symptomatic knee who are not obese and are non-smokers.^44^,^63^ Randomized studies looking at the variants of the ACI technique have been reported from our unit.^48^,^64^ Gooding *et al*. compared the use of the Chondro-Gide membrane with periosteum covering in ACI,^64^ and concluded there to be no statistical difference in using either covering at two years in patients. However, our own experience has shown that patients undergoing periosteum covered ACI, are prone to periosteal hypertrophy of the graft site, and may require arthroscopic shaving of the graft.^37^ As a result, periosteal covered ACI is not favoured in our practice. Another study in our unit by Bartlett *et al*. compared the use of the Chondro-Gide membrane in traditional ACI with the newer MACI technique for OCDs of the knee, and found there to be no significant difference in either method.^48^ However both Bartlett and Basad *et al*. reported MACI being less technically demanding.^49^,^58^ To date, our department has treated in excess of 800 patients who presented with chronic knee pain (pain > six months) and multiple failed previous surgical cartilage repair techniques, with 75-80% showing equivalent or better symptoms compared to their pre-operative state in the short and long-term follow-up following the transplantation of cultured autologous chondrocytes into chondral or OCDs of the knee.

Further developments in this field must now centre on the development of alternative methods including membranes and matrices as well as combination procedures. The use of bone morphogenic proteins, stem cells and gene therapy require investigation.^65^ Future practice must aim at trying to develop a standardized method of ACI in the treatment of cartilage and bony injuries of the joints, with the goal of preventing the development of early onset OA.^66^ Greater long-term follow-up of patients in randomized clinical trails^24^,^37^,^39^,^55^,^57^,^58^ will give a clearer indication as to the therapeutic value of cartilage cell-seeded therapies including ACI and MACI in comparison with other methods of treatment of osteochondral injuries in the knee.
